# Supplementary material for: Balance of Drug Residence and Diffusion in Lacrimal Fluid Determine Ocular Bioavailability in In Situ Gels Incorporating Tranilast Nanoparticles
Source: Pharmaceutics. 2021 Sep 8;13(9):1425. doi: 10.3390/pharmaceutics13091425 (PMC8466670; doi:10.3390/pharmaceutics13091425)
Supplement: Supplementary file 1 [file pharmaceutics-13-01425-s001.zip › pharmaceutics-1365586-supplementary.pdf]

pharmaceutics

# Supplementary Materials: Balance of Drug Residence and Diffusion in Lacrimal Fluid Determine Ocular Bioavailability in In Situ Gels Incorporating Tranilast Nanoparticles

Misa Minami, Hiroko Otake, Yosuke Nakazawa, Norio Okamoto, Naoki Yamamoto, Hiroshi Sasaki and Noriaki Nagai

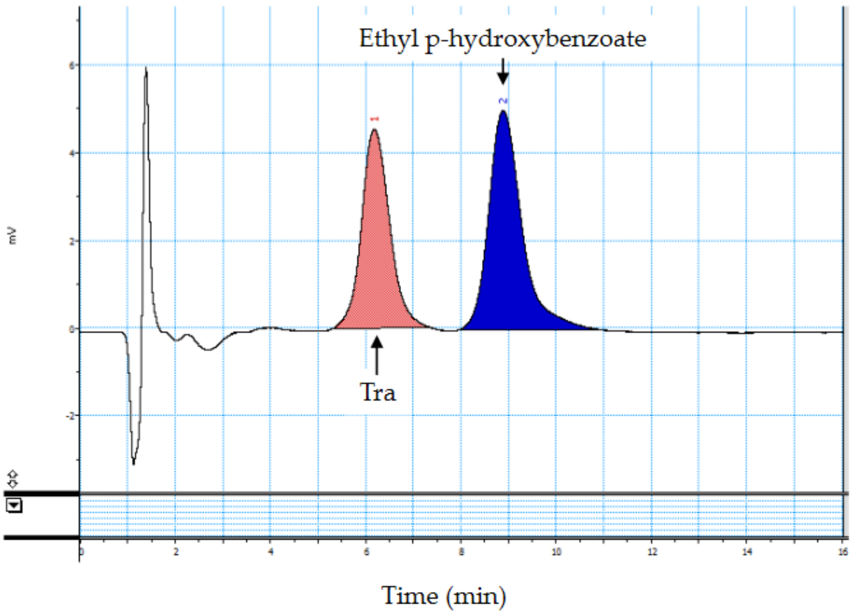


**Figure S1.** Images of the HPLC chromatogram for Tra analysis.
